# PDMS composites with photostable NIR dyes for multi-modal ultrasound imaging

**DOI:** 10.1557/s43580-022-00208-3

**Published:** 2022-01-18

**Authors:** India Lewis-Thompson, Shaoyan Zhang, Sacha Noimark, Adrien E. Desjardins, Richard J. Colchester

**Affiliations:** 1 Department of Medical Physics and Biomedical Engineering, University College London, Gower Street, London, WC1E 6BT UK; 2 Wellcome/EPSRC Centre for Interventional and Surgical Sciences, University College London, Charles Bell House, Foley Street, London, W1W 7TY UK; 3 Materials Chemistry Centre, Department of Chemistry, University College London, 20 Gordon Street, London, WC1H 0AJ UK

**Keywords:** Composite, Polydimethylsiloxane, Organic dyes, Optical absorption, Photostability, Ultrasound

## Abstract

**Abstract:**

All-optical ultrasound (OpUS) imaging has emerged as an imaging paradigm well-suited for minimally invasive surgical procedures. With this modality, ultrasound is generated when pulsed or modulated light is absorbed within a coating material. By engineering wavelength-selective coatings, complementary imaging and therapeutic modalities can be integrated with OpUS. Here, we present a wavelength-selective composite material comprising a near-infrared absorbing dye and polydimethylsiloxane. The optical absorption for this material peaked in the vicinity of 1064 nm, with up to 91% of incident light being absorbed, whilst maintaining lower optical absorption at other wavelengths. This material was used to generate ultrasound, demonstrating ultrasound pressures $> 1$ MPa, consistent with those used for imaging applications. Crucially, long exposure photostability and device performance were found to be stable over a one hour period (peak pressure variation $<10$%), longer than required for standard clinical imaging applications.

**Graphical abstract:**

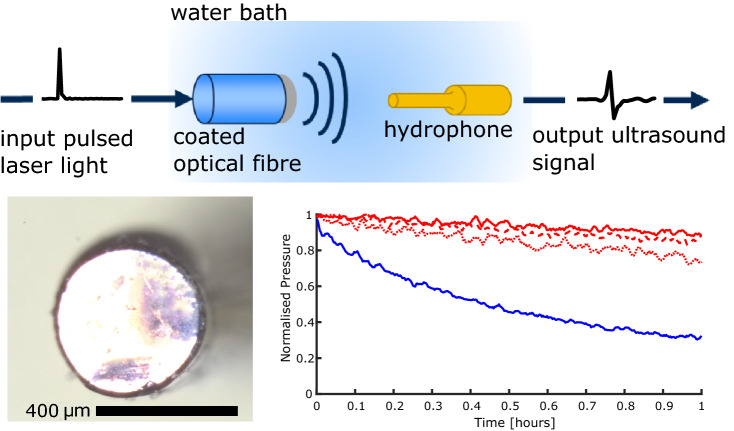

## Introduction

All-optical ultrasound imaging has emerged as an imaging paradigm well-suited for minimally invasive surgical procedures [1–3]. With this modality, ultrasound is generated when pulsed or modulated light is absorbed within a composite coating material [4–8]. Using optical fibres to deliver light for ultrasound generation and reception allows complementary therapeutic and imaging modalities to be integrated to create highly miniaturised devices [1, 4, 9, 10]. One area of interest for these devices is combined imaging and optical ablation, to provide real-time monitoring of ablation lesion depth. However, combining modalities requires carefully engineered composite materials with wavelength-selective absorption [1, 4, 9, 10]. Previous research has demonstrated the use of crystal violet dye or gold nanoparticles embedded in a polydimethylsiloxane (PDMS) host for co-registered ultrasound and photoacoustic imaging [1]. However, these composites suffered from poor photostability under prolonged laser exposure and limited ultrasound pressures and bandwidths, respectively. More recently, quantum dots have been used for co-registered ultrasound and photoacoustic imaging, but the quantum dots were synthesised through a complex process [10].

In this work we introduce a composite comprising a near-infrared absorbing dye and PDMS. The dye (Epolight 9837, Epolin, USA) selected was recommended for absorption of 1064 nm laser light and demonstrates high optical transmission in the visible range when embedded in polycarbonate [11]. This wavelength-selective nature is highly desirable for combining complementary modalities with all-optical ultrasound imaging. Here, we combined the dye with PDMS to fabricate ultrasound generating coatings on optical fibres. Three composites with varying dye concentrations were fabricated to assess the optimal dye concentration; for each concentration 5 ultrasound transmitter repeats were carried out to assess variability. The optical absorption, generated ultrasound, and photostability were characterised and compared to a composite of crystal violet (CV) and PDMS for reference.

## Methods

### Fabrication

Four composite coatings were fabricated; three using Epolight 9837 with varying concentration of dye by weight (2.5, 5, 10 mg) and one using CV for comparison as previous studies show this dye is susceptible to photobleaching [1]. For each composite coating, 5 ultrasound generators were fabricated to test for consistency. All composite coatings were fabricated on the same type of optical fibre, with a fused silica core/cladding and a core diameter of 400 µm (FG400LEP, Thorlabs, UK). The CV fibres were manufactured as per the protocol outlined by Noimark *et al*.  [1]. For the Epolight composites, the selected weight of dye was sonicated at 40 W for 30 s in 0.5 ml of xylene until the dye was dissolved. Subsequently, this solution was manually mixed with 0.25 g of PDMS (MED-1000, Polymer Systems Technology, UK), followed by sonification at 30 W for a total of 30 s, in three 10 s periods with a 10 s rest between.

Optical fibres were prepared for coating by first removing the buffer coating from the distal end of the fibre. Subsequently, the distal end was cleaved manually using a tungsten blade. The prepared optical fibres were manually dipped into the prepared composite solutions and left to cure under ambient conditions for 24 hours with the distal end surface facing up.

**Fig. 1:**
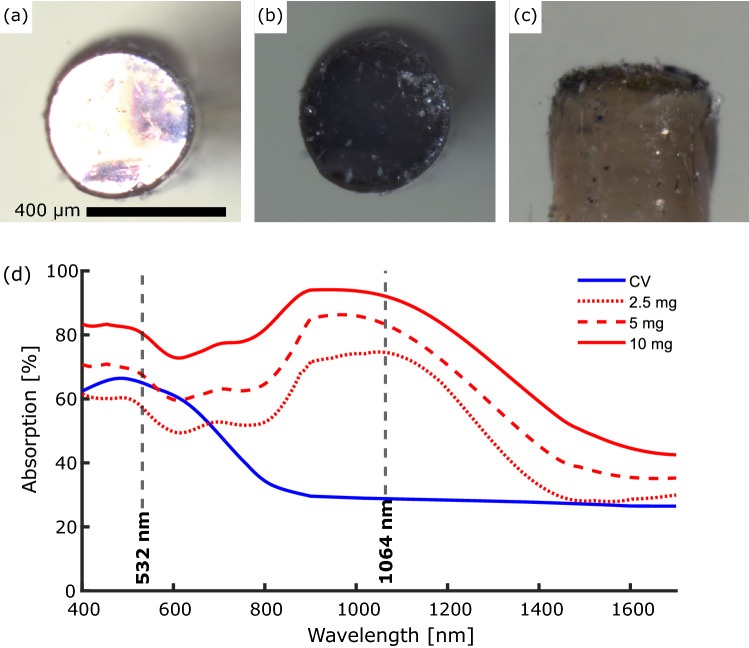
Stereo-microscope images of a 2.5 mg Epolight composite coated optical fibre. (a) End-view with through illumination. (b) End-view with no through illumination. (c) Side-view. Scale bar: 400 µm. (d) Optical absorption spectra for the crystal violet (blue solid line) and Epolight composites (2.5 mg: red dotted line, 5 mg: red dashed line, 10 mg: red solid line).

### Characterisation

Prior to optical and ultrasound characterisation, the coated optical fibres were examined visually using a stereo-microscope (Fig. 1a–c).

The wavelength-dependent optical absorption for the dye-PDMS coated fibres was measured between 400 and 1700 nm using a halogen lamp, an integrating sphere, and two spectrometers (Flame (400–700 nm) & NIRQuest512 (700–1700 nm), Ocean Optics, USA). An uncoated optical fibre was used for reference and a background count was taken for each measurement to remove ambient light and dark counts.

**Fig. 2:**
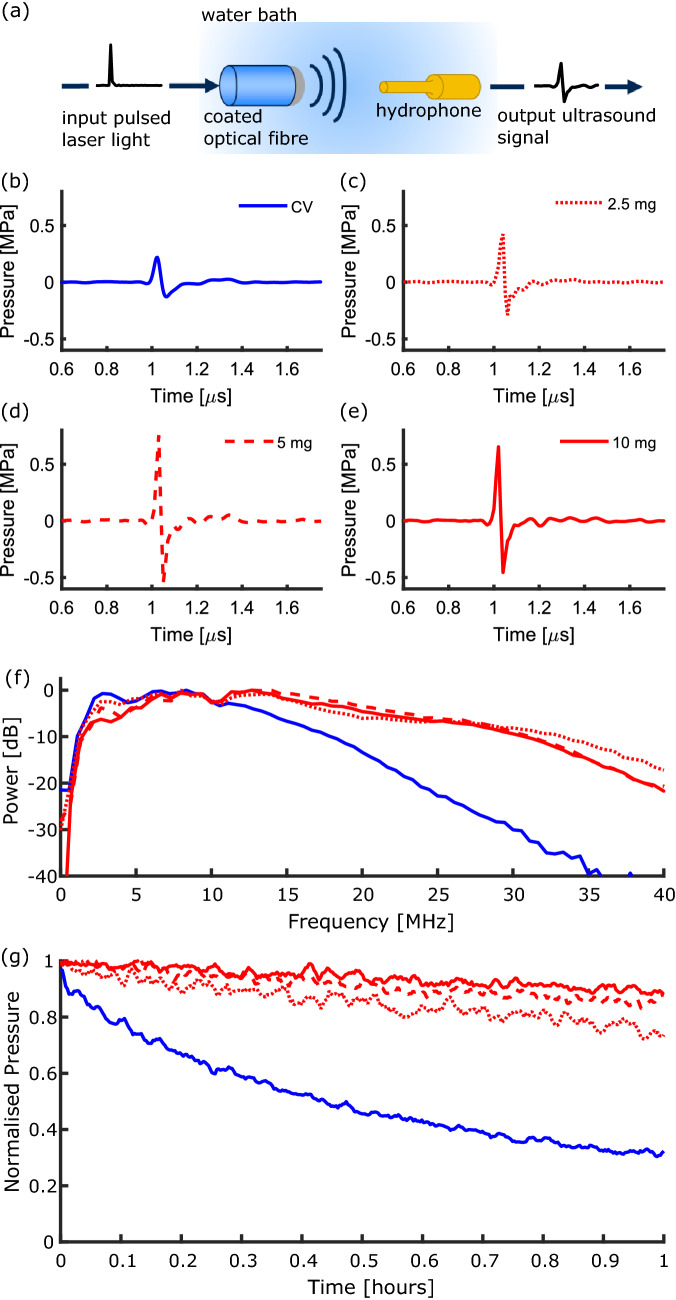
(a) Schematic of the ultrasound characterisation setup with the input laser pulse (pulse width: 2 ns) and the output ultrasound signal. (b)–(e) Ultrasound time-series for the composite coatings (CV coating: blue solid line, Epolight coatings: 2.5 mg-red dotted line, 5 mg-red dashed line, 10 mg-red solid line). (f) Corresponding ultrasound power spectra for the composite coatings. (g) Normalised photostability for the composites compared to the crystal violet composite.

The ultrasound generation was characterised at a pulse energy of 20 µJ (corresponding fluence at coating: 15.9 mJ/cm^2^) using two separate pulsed Nd:YAG lasers for excitation; one with a wavelength of 1064 nm (pulse width: 2 ns, repetition rate: 100 Hz, Spot-10-500-1064, Elforlight, UK), and another with a wavelength of 532 nm (pulse width: 10 ns, repetition rate: 100 Hz, FQ-500-532, Elforlight, UK). The generated ultrasound was measured at a distance of 1.5 mm from the coating using a 200 µm needle hydrophone (Precision Acoustics, UK) with a calibration range 1–30 MHz (Fig. 2a)). To obtain the ultrasound bandwidth, the acquired time-series were Fourier transformed and the hydrophone calibration was applied. To measure the photostability, coatings were continuously exposed to laser pulses for a period of 2 hours and an ultrasound measurement was recorded every 10 seconds.

## Results

### Optical absorption

**Table 1:** Optical absorption and ultrasound generation properties of the composite coatings

	Optical Absorption [%]			Ultrasound Characteristics	
	@ 1064 nm	@ 532 nm	@ 400 - 800 nm	Pressure [MPa]	Bandwidth [MHz]
Crystal violet	$29 \pm 11$	$66 \pm 14$	$51 \pm 15$	$0.31 \pm 0.02$	$10.6 \pm 1.6$
2.5 mg Epolight	$78 \pm 4$	$67 \pm 7$	$65 \pm 8$	$0.46 \pm 0.14$	$16.0 \pm 2.7$
5 mg Epolight	$86 \pm 2$	$73 \pm 5$	$72 \pm 4$	$1.03 \pm 0.13$	$20.7 \pm 3.3$
10 mg Epolight	$96 \pm 2$	$86 \pm 7$	$86 \pm 6$	$0.92 \pm 0.17$	$20.5 \pm 2.3$

The CV and Epolight dyes exhibited different optical absorption profiles (Fig.  1d)). The Epolight composites exhibited an optical absorption peak at *ca.* 900–1100 nm for all dye concentrations (Table 1, Fig.  1d)) with a maximum of $>90$% for the composite with the highest concentration of dye (10 mg). For wavelengths shorter than 900 nm and longer than 1100 nm the optical absorption decreased. The optical absorption at all wavelengths decreased with decreasing dye concentration for the Epolight composites. The CV composite exhibited an absorption peak at *ca.* 532 nm with a value *ca.* 66%. The optical absorption decreased for longer wavelengths to a value $<30$%.

### Ultrasound generation

The Epolight composites exhibited peak-to-peak ultrasound pressure with values up to 1 MPa, with corresponding $-6$ dB ultrasound bandwidths *ca.* 20 MHz (Fig.  2b–f). The generated ultrasound pressure was found to increase with increasing dye concentration from 2.5 mg to 5 mg with maximum values of 0.46 and 1.03 MPa, respectively. For the highest dye concentration of 10 mg, the peak-to-peak pressure was found to decrease slightly, with a value of 0.92 MPa. The CV composite used for reference generated lower peak-to-peak pressure and narrower bandwidths (Table 1, Fig.  2b, f).

### Photostability

The photostability of the Epolight composites was compared to a CV composite (Fig.  2g)). With a laser wavelength of 532 nm at a pulse energy of 20 µJ, the peak-to-peak pressure generated by the CV composite fell by 68% in 1 hour, compared to a reduction by 7% for a 10 mg Epolight composite with a similar optical absorption at 532 nm. All Epolight concentrations demonstrated good photostability at 1064 nm, with the pressure dropping by 25%, 15% and 10% for the 2.5, 5 and 10 mg composites, respectively.

## Discussion and conclusion

In this work we demonstrate the fabrication of a composite comprising an Epolight dye and PDMS and compare it to a CV and PDMS composite. It was found that by reducing the concentration of the Epolight dye, the optical absorption in the range 400 to 800 nm and for wavelengths $>1200$ nm could be reduced whilst maintaining a relatively high optical absorption at 1064 nm for ultrasound generation. The lower optical absorption at wavelengths from 400 to 900 nm and $>1100$ nm is well-suited to a variety of complementary modalities, including photoacoustic imaging and optical ablation. However, whilst the presence of these optical absorption windows are promising for multimodality imaging, further optimisation is needed to reduce the absorption on either side of the peak at 1064 nm. This will be explored in the future work by testing lower concentrations and alternative fabrication methods, such as the bottom-up approach highlighted in [1].

The ultrasound generated by the composites was comparable to that used in previous studies for imaging [2, 3, 8]. All the Epolight composites fabricated outperformed the reference CV composite fabricated here, exhibiting a peak-to-peak pressure $>1$ MPa for composites with 5 mg of dye. It should be noted, however, that previous studies with CV composites have achieved higher pressures and bandwidths [1]. Composites with 2.5 mg generated lower ultrasound pressures than the 5 mg composites, as expected from the corresponding decrease in optical absorption. However, for the higher dye concentration of 10 mg, the peak-to-peak pressure was found to decrease. This may be due to increased ultrasound attenuation within the coating caused by the higher dye loading. Ultrasound bandwidths up to 20 MHz were achieved, sufficient for high resolution ultrasound imaging. However, the lower dye concentration of 2.5 mg exhibited narrower bandwidths than other composites. This could be caused by the longer absorption length, leading to a wider temporal profile in the generated ultrasound pulse.

Crucially, the photostability of the Epolight composites was measured and compared to the CV composite, which has previously been used for *ex vivo* imaging, but demonstrated poor photostability [1]. All of the Epolight composites had acceptable photostability, with the pressure only dropping by *ca.* 10% over a period of 1 hour, compared to a 68% drop for CV composites over the same period. These timescales are relevant for translation of the devices to minimally invasive imaging contexts, where procedures typically take $>1$ h. The results here demonstrate that composite coatings comprising Epolight 9837 are promising for application in multimodality all-optical ultrasound devices. Further work will be carried out to optimise the coatings for specific applications and further improve the generated ultrasound properties. In the future studies, other dyes might be considered with different wavelength selectivities, allowing for application-specific composites. However, for each dye, considerations including the ease of incorporation into the PDMS host, ultrasound generation efficiency and photostability will need to be carefully optimised.

## Data Availability

The datasets generated during and/or analysed during the current study are available from the corresponding author on reasonable request.
